# Effect of Fermented Feed on Growth Performance and Gut Health of Broilers: A Review

**DOI:** 10.3390/ani15131957

**Published:** 2025-07-03

**Authors:** Jim Kioko Katu, Tamás Tóth, Balázs Ásványi, Zoltán Hatvan, László Varga

**Affiliations:** 1 Wittmann Antal Multidisciplinary Doctoral School of Plant, Animal, and Food Sciences, Széchenyi István University, 9200 Mosonmagyaróvár, Hungary; kiokojimmy@gmail.com (J.K.K.); hatvanzoltan@gmail.com (Z.H.); 2 Agricultural and Food Research Center, Széchenyi István University, 9026 Győr, Hungary; 3 Department of Food Science, Széchenyi István University, 9200 Mosonmagyaróvár, Hungary; asvanyi.balazs@sze.hu (B.Á.); varga.laszlo@sze.hu (L.V.)

**Keywords:** fermented feed, broiler growth, gut health, microbiota modulation, poultry nutrition

## Abstract

The fermented feed used in broiler production has gained significant attention for its potential to improve growth performance, enhance gut health, and modulate gut microbiota. This review synthesized findings on the effects of both solid and liquid fermented feed in broilers. Fermentation processes enhance nutrient bioavailability; reduce anti-nutritional factors; and generate beneficial metabolites, such as short-chain fatty acids, which contribute to gut health. Incorporating fermented feed in broiler diets has been shown to improve weight gain, the feed conversion ratio, and nutrient absorption by promoting favorable gut morphology changes, including an increased villus height and villus height-to-crypt depth ratios. Additionally, fermented feed fosters a beneficial microbial environment by increasing lactic acid bacteria populations while reducing pathogenic microbes. Fermentation also modulates gut immunity by regulating cytokine production and stimulating immune cell activity. However, challenges such as inconsistent effects on feed intake and growth during the early production stages underscore the need for optimizing fermentation protocols tailored to broiler production systems. Although the implementation of liquid fermented feed presents logistical challenges, research suggests it can significantly improve feed digestibility. Advances in precision fermentation techniques and multi-strain inoculant use hold promise for further improving fermented feed efficacy. Future research should focus on assessing the long-term impacts, economic viability, and environmental sustainability of fermented feed in commercial poultry systems. Overall, fermented feed offers a promising strategy to enhance productivity and sustainability in broiler farming while reducing the reliance on conventional feed additives. This review reflects the body of knowledge at the time of writing.

## Simple Summary

This review explored how fermented feed can improve the growth and gut health of broiler chickens. Fermentation makes nutrients more digestible and reduces anti-nutritional compounds, supporting better health and performance. It also promotes beneficial gut bacteria, strengthening immunity and reducing harmful microbes. Studies have shown positive effects on weight gain and feed conversion efficiency, though results can vary in young chickens. Overall, fermented feed could reduce antibiotic use and support more sustainable poultry production.

## 1. Introduction

With the global population projected to reach approximately 10 billion by 2050, coupled with ongoing rural-to-urban migration, the demand for food of animal origin is expected to increase significantly [1]. The production of protein sources, such as beef and lamb, derived from cattle, sheep, and goats is facing challenges as critics increasingly highlight ruminants’ role in generating greenhouse gases, especially methane, which exacerbates global warming and contributes to climate change [2]. To address these issues, the increased production of monogastric food animals, such as pigs and poultry, presents a viable alternative. Compared with large and small ruminants, poultry and pigs are generally more efficient and effective at producing animal protein for human consumption [2].

In commercial broiler production, feed expenses represent approximately 75% of the total production costs [2]. Poultry diets primarily comprise cereals, oilseeds, and their by-products, which serve as key sources of energy and protein. Compound feeds are commonly delivered as dry mash or pellets, facilitating transport and storage while streamlining feeding practices and overall poultry house management. Compared with conventional feed additives, fermented feed production offers greater advantages in improving the feed quality, growth performance, and overall poultry health [3,4]. Current developments in fermentation technology are increasingly focused on both solid-state and liquid fermentation methods for feed production [5]. Studies have demonstrated that fermentation effectively reduces anti-nutritional factors (ANFs), enhances the bioavailability of macro- and micro-nutrients, improves the feed digestibility, and stimulates the formation of beneficial bioactive metabolites [6].

Despite these advantages, the application of feed fermentation has largely been restricted to forages for ruminants (i.e., cattle, sheep, and goats), owing to the distinct anatomical and physiological differences in their digestive systems compared with monogastric animals. Studies have demonstrated that fermentation can break down indigestible complexes, such as cellulose and hemicellulose, into simpler, digestible molecules, and this has contributed to the widespread adoption of wet fermented feed in pig production [7]. Beyond its nutritional benefits, fermented feed often contains high concentrations of lactic acid bacteria (LAB), depending on the substrate and fermentation duration [8]. These bacteria lower the pH of the feed and the gastrointestinal environment, thereby modifying the gut microbiota through pathogenic organism suppression via competitive exclusion. This microbial shift contributes to improved intestinal health and overall animal well-being [7]. These advantages have spurred the growing interest in the application of fermented feed in broiler production systems [9].

Previous research on fermented feed in poultry has primarily focused on the solid-state fermentation of individual raw materials, particularly grains, rather than complete feeds. Few studies have explored liquid fermented feed use in poultry production, even though the fundamental principles of feed fermentation are similar for both solid- and liquid-state processes.

Based on the currently available information, this review aimed to evaluate the effects of both solid and liquid fermentation—applied to individual feed ingredients or complete compound feeds—on the growth performance, gut microbiota composition, and gut health in broiler chickens.

## 2. Enhancing Broiler Growth Performance Through Fermented Feed: Nutritional and Productivity Insights

Optimal broiler performance is influenced by several factors, including genetics, environmental management, health, and nutrition. Advances in nutritional research and diet formulation have significantly enhanced broiler productivity [2]. Fermented feed use has shown a positive impact on pig nutrition and productivity by reducing ANFs, improving feed digestibility, and enhancing gut health [10]. Similarly, several studies have reported growth and health benefits in broilers when diets include one or more fermented ingredients [11]. Most of these studies focused on incorporating either solid-state fermented or re-dried liquid-state fermented individual ingredients into broiler diets, with the aim of replicating the production benefits observed in pigs fed liquid fermented feed. Re-dried, soaked feeds have been shown to enhance nutrient utilization, especially proteins [12]. Studies have demonstrated that mixing conventional feeds with sufficient water to form a broth-like consistency can increase the protein retention time in the gut and, in some cases, even stimulates feed intake (FI), allowing for the more efficient utilization of feed with a low nutrient density [13]. Some of the major effects of fermented feeds on broiler chicken growth performance are summarized in Table 1.

Broilers supplemented with *Saccharomyces cerevisiae*-fermented de-oiled rice bran, either alone or fortified with urea or wheat flour, exhibited significantly (*p* < 0.05) higher total weight gains (WGs) of 1235, 1236, and 1255 g, respectively, compared with 1139 g in the control group fed unfermented de-oiled rice bran [22]. Although the FI did not differ significantly between the fermented and control groups, the secretion of yeast-derived enzymes during fermentation enhanced the bioavailability of nutrients, such as peptides, sugars, and minerals. Specifically, baker’s yeast (*Saccharomyces cerevisiae*) produces enzymes like phytases and cellulases, which degrade phytates and fiber, respectively [23]. Additionally, the groups supplemented with fermented de-oiled rice bran, whether alone or fortified with urea or wheat flour, exhibited significantly (*p* < 0.05) lower feed conversion ratios (FCRs) of 1.75, 1.74, and 1.73, respectively, compared with 1.88 in the control group (*p* < 0.05) [22].

The increase in microbial populations during fermentation has been shown to elevate the microbial protein concentration within the fermented substrate, thereby enhancing the feed’s probiotic effects on the host’s gastrointestinal tract [22]. In a study that evaluated broilers fed unfermented versus fermented feed based on a corn–soybean meal (SBM) diet, in either mash or pellet form, a two-stage fermentation strategy was employed using *Bacillus* spp. in both stages or *Bacillus* spp. in the first stage followed by various *Lactobacillus* spp. in the second stage. Notably, birds fed the mash diet fermented with only two *Bacillus* strains (*Bacillus subtilis* var. *natto* N21 in stage one and *Bacillus coagulans* L12 in stage two) exhibited a significantly improved FCR of 1.33 (*p* < 0.05), reflecting a 7% enhancement compared with the control group that received unfermented feed, which had an FCR of 1.43 [15]. By day 21, the birds fed pelleted or unpelleted fermented feed achieved significantly higher WGs of 792 and 781 g, respectively, compared with 741 g in the control group fed unfermented pellets. Similarly, by day 35, the broilers fed two-stage fermented pelleted feed using *Bacillus* strains alone achieved a significantly (*p* < 0.05) higher final weight of 1981 g compared with 1858 g in the control group [15]. The resilience of *Bacillus* species to high temperatures during the drying and pelleting processes likely contributed to the higher retention and concentration of *Bacillus subtilis* and *Bacillus coagulans* in birds fed dry or pelleted fermented feed, which enhanced the probiotic effects and promoted an improved growth performance [15].

To assess the effects of partially replacing a corn–SBM basal diet with *Bacillus*- or *Saccharomyces*-fermented distillers’ dried grains with solubles (DDGS), broilers were divided into six groups. The control group received the basal diet, the negative control was supplemented with unfermented DDGS, and the remaining four treatment groups were supplemented with either *Bacillus subtilis*- or *Saccharomyces cerevisiae*-fermented DDGS, with or without the addition of a multienzyme blend (containing endo-1,4-β-xylanase, subtilisin, and α-amylase). Between days 25 and 36, the birds fed *Bacillus*- or *Saccharomyces*-fermented DDGS without enzyme supplementation exhibited significantly (*p* < 0.05) lower FCRs of 1.44 and 1.48, respectively [24]. Enzyme supplementation further improved the FCRs to 1.42 for *Bacillus*-fermented DDGS and 1.59 for *Saccharomyces*-fermented DDGS compared with 1.60 in the control group and 1.53 in the negative control group. However, the partial replacement of the basal diet with fermented DDGS did not significantly affect (*p* > 0.05) the body weight (BW), WG, or FI across all the treatment groups [24].

ANFs, such as phytates and a high fiber content, typically limit the inclusion of whole wheat grains in broiler diets to a maximum of 30% [25]. However, the incorporation of *Lactobacillus* strain AD2-fermented whole wheat or barley at inclusion levels of 0, 20, 40, and 60% did not result in any adverse effects on the FI, daily WG, or overall growth performance. The fermented wheat and barley inclusion at these levels demonstrated a significant (*p* < 0.05) linear increase in broiler weight after 14 days, with coefficients of determination (*R*^2^) of 0.5 and 0.6 for fermented whole wheat and barley, respectively [25].

To assess whether rapeseed meal (RSM) fermented with *Aspergillus niger* PTCC5010, *Lactobacillus acidophilus* PTCC1643, and *Bacillus subtilis* PTCC1156 could mitigate *Salmonella* infections in broilers, a study evaluated diets in which 50% of the SBM in a corn–SBM control diet was replaced with fermented RSM. The broilers fed the fermented RSM diet exhibited significantly (*p* < 0.05) higher WGs of 2233 g compared with those fed diets that contained 50 or 100% unfermented RSM, which resulted in WGs of 2107 and 1796 g, respectively [20]. The birds fed the control diet did not have a statistically different FCR (1.93) compared with those supplemented with 50% fermented RSM (1.94), although this FCR in birds supplemented with 50% fermented RSM was significantly (*p* < 0.05) lower compared with those on 50 or 100% RSM (2.06 and 2.37, respectively). By the end of the 42-day experiment, replacing 100% of the SBM with fermented RSM significantly (*p* < 0.05) increased the FCR to 2.08 compared with the control diet (1.93), despite no significant differences in the FI across all groups [20]. The reduced WG and higher FCR in birds fed 50 and 100% unfermented RSM can be attributed to several ANFs, including phytates, phenolic compounds, high crude fiber content, reduced protein digestibility relative to SBM, and the lysine–arginine amino acid imbalance associated with high RSM inclusion levels [17,20].

A separate study investigated the effects of fermented RSM on growth, nutrient digestibility, intestinal ecology, and intestinal morphology in broilers. Birds were fed either a control corn–SBM diet alone, a diet supplemented with 10% unfermented RSM, or a diet supplemented with 10% solid-state fermented RSM using mixed cultures of *Limosilactobacillus fermentum*, *Enterococcus faecium*, *Saccharomyces cerevisiae*, and *Bacillus subtilis* [16]. Broilers supplemented with 10% fermented RSM exhibited a significantly (*p* < 0.05) higher WG and lower FCR from days 1 to 42 compared with those fed 10% unfermented RSM. However, there were no significant (*p* > 0.05) differences in the growth performance between the broilers supplemented with 10% fermented RSM and those fed the control corn–SBM diet alone [16].

Another study evaluated the effects of supplementing conventional broiler feeds with dry fermented feed at inclusion levels of 0, 10, 15, 20, and 25%, or with 10% wet fermented feed, on the growth, immunity, and gut microbiota [26]. The birds fed 15% dry fermented feed achieved a significantly (*p* < 0.05) higher final BW of 2.16 kg, compared with 2.08 kg in the control group. From days 22 to 42, the feed intake was significantly (*p* < 0.05) higher in the birds supplemented with 10 and 15% dry fermented feed, with 2.35 and 2.40 kg, respectively, compared with those on 20 and 25% dry fermented feed and the control, with 2.25, 2.24, and 2.24 kg, respectively. No significant differences were observed in the FCR during the starter (days 1–21), grower (days 22–42), and entire (days 1–42) periods across all the groups. The birds supplemented with 10% wet fermented feed exhibited an increased FI of 3.3 kg over the entire experimental period (days 1–42), compared with 2.13 kg (days 21–42) and 3.1 kg (days 1–42) in the control group [26].

Overall, the incorporation of fermented feed ingredients into broiler diets has demonstrated variable but generally positive effects on growth performance, including an improved FCR, increased WG, and enhanced nutrient utilization. The efficacies of these outcomes were influenced by several factors, such as the type of fermentation (solid- versus liquid-state), the inclusion level, and the specific microbial inoculants employed. Moderate inclusion levels combined with well-characterized microbial strains tended to produce the most consistent benefits. Nevertheless, the variability across the studies highlights the need for standardized fermentation protocols and further investigation into strain-specific effects and underlying action mechanisms.

### Effects of Wet and Liquid Fermented Feeds on Broiler Growth Performance

In commercial poultry production, providing dry feed in the form of mash or pellets is the conventional and widely accepted practice. Research on the use of liquid fermented feed in broilers is limited, partly due to concerns about its suitability for birds and the potential risk of wet litter [10]. Additionally, large-scale wet mash application is often discouraged because of logistical challenges and reports suggesting no significant nutritional advantages [13]. Despite these challenges, broilers show a natural preference for wet feed over dry feed. Studies have reported that wet feeding can enhance the daily WG and FI. In fact, some evidence suggests that broilers may find it difficult to consume enough dry mash to fully realize their genetic growth potential [27,28,29]. Research has highlighted several benefits of wet feeding in broilers, including improvements in dry matter intake, growth rate, and FCR [13,27,30,31].

Research also indicates that re-drying soaked or liquid fermented grains may not be necessary to fully harness the fermentation benefits. Soaking cereals has been demonstrated to enhance their nutritional value for inclusion in broiler diets [32]. The improved digestion observed with wet feeding is attributed not only to the activation of endogenous grain enzymes but also to the greater ease and speed with which digestive juices penetrate the substrate [13].

In tropical regions, particularly in savannah ecological zones, diurnal temperatures often range from 28 to 42 °C, exceeding the thermo-neutral zone of 26 °C that is ideal for commercial poultry production. Under such high ambient temperatures, heat stress can impair the FI and suppress growth performance in broilers [33]. Modern broiler breeds, characterized by high metabolic rates, are particularly susceptible to heat stress due to their high performance [34]. While environmental temperature control within poultry housing is a common heat stress management strategy, other measures, such as wet feeding, have received less attention. However, studies suggest that wet feeding may improve the FI and growth performance under heat stress conditions [30,31].

In a study that evaluated the effect of dry versus wet feeding on broiler growth from days 28 to 56, broilers fed a wet feed alone showed a significant (*p* < 0.05) increase in FI (174 g/day) compared with those fed dry feed alone (152 g/day) [33]. The birds fed a combination of wet and dry feed also had a higher FI (166 g/day) than those on dry feed alone [33].

In response to heat stress, broilers typically reduce activities, such as walking and feeding, and spend the most time acclimatizing to the conditions by resting, panting, and spreading their wings to minimize metabolic heat [35]. Wet feeding can help reduce or eliminate these heat stress responses in birds reared under high environmental temperatures. Birds on wet feed or a combination of wet and dry feed showed a significantly (*p* < 0.05) higher average daily WG of 64 g/day compared with 58 g/day in birds on dry feed alone [33]. Although the FCR remained consistent across all treatment groups, the birds fed wet feed or wet plus dry feed achieved a heavier mean final live weight (1.8 kg) compared with those fed on dry feed alone (1.63 kg). Adjusting the feed consumption for all groups to achieve a 1.8 kg market weight, wet feeding demonstrated improved efficiency in meat production [33].

Another study assessed the growth performance of broilers fed either dry or wet (grain-to-water ratio of 1:1.3) wheat-, barley-, or oat-based diets from days 28 to 42 [32]. The birds on wet cereals showed significantly (*p* < 0.05) higher daily FIs of 130.7, 128.3, and 139.7 g for wet wheat, barley, and oats, respectively, compared with 97.8, 99.1, and 107.1 g for their dry counterparts. The weekly BW gain during the same period was also significantly (*p* < 0.05) higher in the birds fed soaked wheat and oats at 489.2 and 442.5 g per week, respectively, compared with 385.5 and 347.5 g per week for the birds on dry wheat and oats. From days 7 to 21, the birds fed soaked wheat and oats also demonstrated significantly (*p* < 0.05) higher daily FIs of 82.2 and 74.9 g, respectively, compared with 68.0 and 53.6 g for birds fed dry wheat and oats. Despite these proportional increases in FI and BW gains, the feed conversion efficiency was unaffected, showing no significant differences between the groups [32].

Cereals and cereal by-products commonly used in poultry diets are rich in non-starch polysaccharides, which increase the digesta viscosity and reduce digestibility within the small intestine. However, soaking or wetting grains significantly (*p* < 0.05) lowers the digesta viscosity in the gastrointestinal tract (GIT), which speeds up the digesta passage and enhances nutrient absorption. These effects are especially notable in the small intestine and contribute to improved feed utilization [32].

A study assessed broiler performance when fed either dry or wet feed (using feed-to-water ratios of 1:1.5, 1:1.75, 1:2.0, and 1:2.25) from days 28 to 49. The broilers on wet feed exhibited significantly higher average daily FIs (183, 185, 182, and 171 g, respectively), daily BW gains (80.0, 75.8, 79.0, and 77.1 g), and carcass weights (1.72, 1.72, 1.68, and 1.68 kg) compared with those on dry feed, which had an average daily FI of 147 g, daily BW gain of 52 g, and final carcass weight of 1.14 kg [12].

In another 28-day study that evaluated the growth performance of broilers fed commercial diets in dry, wet, or fermented forms, the birds on fermented feed exhibited a significantly (*p* < 0.05) higher total FI (3.2 kg) and final BW (1.2 kg) compared with those on dry feed (2.8 and 1.0 kg, respectively). Furthermore, the birds fed liquid diets (wet or fermented) demonstrated significantly (*p* < 0.05) better FCRs of 2.72 and 2.78, respectively, compared with the birds on dry feed (FCR of 2.9) [36].

The enhanced fermented feed nutritional characteristics, such as improved palatability and digestibility, an increase in beneficial gut microbes (e.g., LAB), a higher microbial enzyme concentration, and ANF degradation, are among the key mechanisms contributing to the improved growth performance in broilers fed fermented diets [11,37].

Additional factors specific to wet and/or liquid fermented feed, including changes in intestinal morphology, also significantly influence broiler growth performance [38]. Alterations in the intestinal histomorphology can affect the intestinal surface area and increase the capacity for nutrient absorption. The integrity of the small intestine is maintained by a delicate balance between the proliferation and differentiation of enterocytes [39]. For instance, birds supplemented with a probiotic *Lactobacillus acidophilus* strain showed significantly (*p* < 0.001) improved 21-day WG (643 g) compared with control birds (600 g) [40]. These birds also exhibited a significantly (*p* < 0.05) higher villus height (VH) (962 µm) and villus height-to-crypt depth (VH/CD) ratio (4.5) compared with the control group (802 µm and 4.3, respectively) [40]. Similarly, feeding moist fermented diets to broilers during the finisher period significantly (*p* < 0.05) increased the VH in the mid-jejunum and mid-ileum by 23 and 16%, respectively, compared with broilers on dry unfermented feed [10].

Increases in the VH and VH/CD ratio enhance nutrient absorption within the small intestine [40]. To evaluate the effects of supplementing broiler diets with *Aspergillus niger*-fermented or unfermented *Ginkgo biloba* leaves, birds were fed increasing levels of fermented leaves (FR1, FR2, and FR3) at starter and grower inclusion rates of 0.2 and 0.4%, 0.35 and 0.7%, and 0.5 and 1.0%, respectively [41]. The birds that received fermented diets had significantly (*p* < 0.05) higher duodenal VHs of 1.69, 1.68, and 1.68 µm, respectively, compared with the control (1.56 µm) and unfermented groups (1.63 µm). The jejunal VH was also significantly (*p* < 0.05) greater in FR2 (1.3 µm) compared with the control (1.1 µm) and unfermented groups (1.2 µm). Additionally, the VH/CD ratios in FR2 and FR3 were significantly higher in the duodenum (7.87 and 8.10), jejunum (7.32 and 7.06), and ileum (8.67 and 8.18) compared with the control (7.25, 5.92, and 7.51, respectively) [41].

However, wet fermented feed use may have adverse effects on broiler performance during the starter and grower phases. Broilers fed moist fermented diets exhibited a significantly (*p* < 0.05) reduced FI and WG by 40 and 44%, respectively, during the starter period, and by 23 and 16%, respectively, during the grower period [10]. Variability in the water absorption capacity between different feed ingredients can lead to inconsistencies in the liquid feed texture; excessive moisture content may inhibit the feed intake and contribute to the leaching of essential nutrients, such as amino acids, vitamins, and minerals [13]. Furthermore, liquid feed may promote selective feeding, with birds preferring larger feed particles, which can result in an uneven nutrient intake. High moisture levels can also lead to wet litter, negatively affecting cleanliness and bird welfare, while increased susceptibility to mold growth during storage may pose additional health risks [13].

Wet or moist feed tends to be bulky and less attractive to chicks after soaking, which can reduce the FI. Broiler chicks may require time to adapt to such feed, resulting in lower dry matter intake during the adaptation period [10].

Additionally, high concentrations of organic acids, such as acetic and lactic acids (>286 mmol/L), produced during fermentation can reduce the feed palatability and subsequently decrease the FI. Nutrient depletion, including the loss of low-molecular-weight sugars and amino acids due to microbial degradation or leaching into the water phase of liquid fermented feed, may adversely affect broiler growth, particularly during the starter and grower phases [10]. To mitigate these negative outcomes, it is critical to maintain an appropriate water-to-feed substrate ratio; select an effective inoculum; and carefully control the fermentation parameters, such as duration, temperature, and pH.

Similar to solid-state fermentation, in vitro studies have demonstrated that wet fermented feed enhances the organic matter and crude protein digestibility [14]. The liquid fermentation effects are dependent on the metabolic activity and characteristics of the microbial inoculum used [5]. Nevertheless, feeding broilers fermented diets has been shown to improve nutrient utilization beyond the benefits associated with providing wet feed alone, ultimately contributing to enhanced productivity [14].

Overall, wet and fermented feeds have the potential to enhance broiler performance by promoting intestinal development, increasing the VH, and improving the nutrient absorption and digestive enzyme activity. These benefits are mediated by microbial metabolites produced during fermentation, such as organic acids and enzymes, which contribute to improved digestive efficiency and gut morphology. However, unbalanced fermentation processes or excessive moisture content can negatively affect the FI and overall performance, underscoring the importance of precise control over fermentation parameters and feed consistency to fully realize the benefits of fermented feeding strategies in broiler production.

## 3. Enhancing Broiler Gut Health Through Fermented Feed: Immunity, Morphology, and Microbial Composition

Gut health in most animals, including poultry, encompasses the interaction between gut immunity, gut morphology, physiology, and gut microbial composition [42]. The correlation between gut health and growth performance in food animals is widely recognized, with the small intestine playing a critical role as the primary site for digestion and nutrient absorption [43].

The immune system in poultry is broadly categorized into two main components: the central immune system [thymus, bone marrow, and bursa of Fabricius (BoF)] and the peripheral immune system (spleen and cecal tonsils). Lymphocytes are generated in the central immune organs and subsequently migrate to peripheral sites, where they mature and contribute to the development of gut-associated lymphoid tissue (GALT). In broilers, gut microbiota-driven antigenic stimulation is the key to activating intestinal immunity, while maternal antibodies and innate immunity are crucial for early disease resistance during their rapid growth cycle [44].

Nutritional interventions, such as the inclusion of fermented feeds, probiotics, and prebiotics, have been shown to promote the effective development of the innate immune system in broilers [45]. Several studies have demonstrated that probiotics, prebiotics, bioactive compounds (e.g., organic acids, bacteriocins), and the reduction in ANFs and immunogens (conglycinin, β-conglycinin) in fermented feed enhance both innate and adaptive gut immunity in broilers [26,45,46,47]. These effects are mediated through various mechanisms, including (1) gut microbiota modification, (2) gut morphology alterations, (3) immune cell regulation, (4) immune signaling molecule [e.g., chemokines, interferons (IFNs), and cytokines] modulation at the cellular and gene levels [11].

### 3.1. Fermented Feed’s Effect on Intestinal Immune Cell Signaling

The modulation of gut mucosal immunity requires the detection of pathogen-associated molecular patterns by host pattern recognition receptors, such as toll-like receptors (TLRs). These plasma membrane-bound receptors, upon stimulation by antigens, initiate a cascade of events that activate transcription factors, including the interferon regulatory factor and the nuclear factor kappa B [48]. These factors translocate to the nucleus to drive the transcription and translation of RNA involved in the synthesis of pro-inflammatory cytokines [49].

Cytokines are peptides produced by immune cells, including T-cells, B-cells, macrophages, endothelial cells, and fibroblasts, which act as signaling molecules to activate and regulate other immune cells during inflammation [50]. TLRs, expressed by mononuclear phagocytes on the basolateral surfaces of intestinal epithelial cells (IECs), recognize microbial antigens, such as bacterial cell wall lipopolysaccharides, and initiate immune signaling cascades [51]. This recognition event triggers the release of pro-inflammatory cytokines, notably interleukin (IL) 6, IL-1β, and tumor necrosis factor (TNF), which serve to recruit immune effector cells, including natural killer cells, CD8^+^ T-cells, and macrophages, to the site of infection, thereby promoting gut inflammation [49,51].

Studies have demonstrated that fermented feed can improve the intestinal morphology by attenuating excessive inflammation [10]. To investigate the effects of a solid-state fermented feed additive (FFA), which comprised corn, SBM, and wheat bran mixed with 5% yeast extract and 5% molasses and was fermented with *Lacticaseibacillus casei* at 37 °C for 24 h, on the small intestine morphology, immunity, and gut microbiota, broilers were fed four diets: a basal diet as a negative control (NC), a basal diet supplemented with antibiotics as a positive control (PC), and two experimental diets with the basal diet supplemented with FFA at a low dose (FFL, 0.3 kg/t) or high dose (FFH, 3 kg/t) [45]. Gene expression analysis revealed that the anti-inflammatory cytokine IL-10/β was significantly (*p* < 0.05) upregulated in the FFL and FFH groups, which showed 2.1-fold and 1.7-fold increases, respectively, compared with the negative control group. However, the IL-10/β expression was similar between the PC, FFL, and FFH groups. There were no significant (*p* > 0.05) differences in the IL-6/β, IFN-γβ/β, and IL-4/β expressions across all four groups [45]. IL-10 is a potent anti-inflammatory cytokine and is responsible for the immune tolerance of the gut against intestinal microbiota [52,53]. The low pathogenic microbe concentration in fermented feed reduces the exposure of the intestinal tract to pathogens, thereby mitigating excessive or sustained gut inflammation [10]. This effect helps maintain a healthy intestinal morphology and enhances the gut immunity [45].

Various strains of LAB, particularly those belonging to the genera *Lactobacillus*, *Pediococcus*, and *Streptococcus*, are commonly used as starter organisms in feed fermentation [54]. Lactobacilli, a significant component of human and animal microbiota, are prevalent in the digestive and female reproductive systems. *Lactobacillus* strains are among the most commonly used probiotics in the food and feed industries, contributing to the probiotic effects of fermented feed. The antibacterial activities of lactobacilli and the organic acids produced during fermentation have been extensively documented [55]. Moreover, fermented feed’s immune-regulatory effects are closely linked to the probiotic strains used as inoculants, particularly *Lactobacillus* spp. [56]. Feed fermentation not only reduces or transforms ANFs into less harmful forms but also enhances the concentration of beneficial probiotics, enzymes, and bioactive metabolites [57,58]. ANFs are known to impair digestive function and nutrient absorption, which can negatively influence immune organ development [45].

Fermented feed has been shown to improve gut morphology by reducing excessive inflammation within the gastrointestinal tract [10]. Feeding broilers LAB-fermented feed, which typically contains high LAB concentrations, has been associated with enhanced disease resistance, although the underlying immunomodulatory mechanisms remain incompletely understood [45]. It was hypothesized that LAB in fermented feed may influence cytokine production, thereby modulating immune responses [59]. Emerging evidence suggests that certain *Lactobacillus* strains may downregulate TLR4 mRNA expression, contributing to anti-inflammatory effects.

Dietary supplementation with fermented plant products, such as fresh vegetables fermented using various *Lactobacillus* strains, has been shown to modulate gut microbiota, enhance intestinal immune function, and strengthen the intestinal barrier [56]. Similarly, the inclusion of *Lacticaseibacillus casei*-fermented SBM in broiler diets has been found to upregulate the mRNA expression of interleukins in the ileal mucosa, indicating an activated mucosal immune response [15].

The probiotic effects of LAB in fermented feed also contribute to immunomodulation within the GIT, particularly through the competitive exclusion of microbial pathogens during the early life stages [60]. Treatment with *Lactobacillus* spp. has demonstrated immunostimulatory activity in chickens’ intestinal mucosa [61]. Furthermore, the presence of lactobacilli in the gut of poultry has been linked to enhanced immunoglobulin A synthesis, a response believed to be mediated by short-chain peptides released during microbial metabolism [62].

The immune organ index is commonly used to reflect the levels of immune response in broilers. The weight of the BoF, an avian-specific immune organ, has been shown to be influenced by exogenous feed [26]. Fermented feed has been reported to increase the relative BoF weight in broilers, likely due to the high concentration of beneficial microorganisms, particularly bifidobacteria [63]. Dietary supplementation with *Lactiplantibacillus plantarum*, *Bacillus subtilis*, and *Saccharomyces cerevisiae* using dried fermented feed at inclusion levels of 10–25% significantly (*p* < 0.05) increased the BoF index (1.4–2.7 g/kg) compared with the control group (1.0 g/kg) [26]. These enhancements were accompanied by significant (*p* < 0.05) increases in serum immunoglobulin concentrations, including IgA (6.4–7.3 vs. 6.4 µg/mL), IgG (66–89 vs. 65 µg/mL), and IgM (4.0–5.7 vs. 3.8 µg/mL) in the treatment groups [26].

Short-chain fatty acids (SCFAs), including acetic, propionic, and butyric acids, along with lactic acid, are major end-products of the anaerobic fermentation of indigestible dietary fibers by commensal gut bacteria, primarily in the cecum [64]. SCFAs serve as energy sources for IECs and regulate water and electrolyte absorption. Numerous studies have demonstrated that SCFAs influence gut health by modulating the composition and metabolic activity of gut microbiota, serving as an essential link between intestinal microbes and overall gut health [65]. In a study that evaluated healthy broilers, SCFA concentrations in the ileum and cecum were found to increase linearly with age (*p* < 0.05), correlating with improvements in the gut morphology and microbial stability [66]. SCFAs also exhibit immunostimulatory properties, priming immune cells and enhancing host immune responses when included in poultry diets [67].

IECs, as the primary source of gut cytokines, are influenced by contact with SCFAs. Studies have shown that SCFAs regulate the synthesis and production of these immune-signaling molecules [18,65,68,69]. SCFAs also modulate leukocyte function, which subsequently affects the production of cytokines, such as TNF-α, IL-2, IL-10, and various chemokines [65]. Additionally, SCFAs can directly regulate macrophage activity, influencing the production of cytokines, including TNF-α, IL-1β, and IL-6 [70].

SCFAs inhibit histone deacetylase, thereby regulating the gene expression of cytokines in leukocytes and endothelial cells. SCFAs promote the acetylation of both histone and non-histone proteins, influencing the transcription of various cytokines. Notably, butyrate has been shown to attenuate histone deacetylase activity, leading to increased anti-inflammatory cytokine IL-10 production [71]. Both propionate and butyrate have demonstrated a capacity to inhibit TNF-α expression [65]. However, in vitro studies have also shown that lymphocyte incubation with butyrate can significantly (*p* < 0.05) reduce IL-2 and IL-10 production [70], suggesting a complex, context-dependent immunomodulatory role.

*Ganoderma lucidum*, commonly known as “reishi” in Japan, is a macro-fungus historically used in traditional herbal medicine [72]. In a study that assessed the physicochemical properties of *Ganoderma lucidum* oligosaccharides and their effects on intestinal microbiota following fermentation, intestinal content inoculation with *Ganoderma lucidum* oligosaccharides resulted in a significant (*p* < 0.05) increase in SCFA concentrations compared with controls, indicating a beneficial impact on the gut microbial composition [73].

Serum cytokine levels serve as reliable indicators of humoral immune responses in broilers [74]. Pro-inflammatory cytokines, such as IFN-γ, have been associated with enhanced disease resistance [75], while TNF-α plays key roles in regulating inflammation, immune organ development, and lymphocyte homeostasis [74]. In a study that evaluated the effects of fermented *Ganoderma lucidum* supplementation on broiler growth and immune responses, birds that received fermented *Ganoderma lucidum* showed significantly (*p* < 0.05) elevated serum levels of pro-inflammatory cytokines, including IFN-γ, IL-1, IL-2, IL-12, and TNF-α, along with a reduction in the anti-inflammatory cytokines IL-4 and IL-10, relative to birds that received unfermented *Ganoderma lucidum* [74].

Fermented feed further contributes to gut immunity by reducing the concentrations of ANFs and immunogenic proteins in the intestinal tract. SBM, for instance, contains allergenic proteins, such as glycinin and β-conglycinin, which are recognized gut allergens capable of inducing transient hypersensitivity and intestinal inflammation, particularly in piglets [76,77]. The microbial fermentation of SBM using fungi (e.g., *Rhizopus*, *Aspergillus*) or bacteria (e.g., *Bacillus*, *Lactobacillus*) has been shown to significantly (*p* < 0.05) reduce the levels of these allergenic proteins, thereby enhancing the nutritional and immunological safety of SBM-based feeds [78].

### 3.2. Fermented Feed’s Effect on Immune Cell Dynamics in the Gastrointestinal Tract

Various lactobacilli strains are known to colonize the poultry GIT, modulating the gut microbiota and promoting immunity and overall gut health [60]. Studies in humans have demonstrated that lactobacilli can influence immune responses by modulating cytokine activity, which, in turn, regulates T-lymphocyte activity, particularly CD4^+^ cells [79]. The high concentration of LAB in fermented feed lowers both the feed and gut pH values, creating an environment unfavorable for pathogenic microbes, such as *Salmonella* and coliform bacteria. Additionally, the presence of *Lactobacillus* spp. in the gut contributes to maintaining a balanced gut microbiota, which is essential for supporting immunity and health in poultry [80].

A study that investigated leukocytic infiltration (CD3^+^, CD4^+^, and CD8^+^ cells) in the intestinal epithelium and lamina propria of broiler chicks infected with *Salmonella enterica* subsp. *enterica* serovar Enteritidis (*Salmonella* Enteritidis) provided key insights [60]. Treatment with lactobacilli strains or cecal microbiota, with or without exposure to *Salmonella* Enteritidis, significantly (*p* < 0.05) increased the leukocyte infiltration in the duodenum, jejunum, and cecum of 12-day-old chicks. The intestinal epithelial leukocytic infiltration predominantly comprised CD3^+^ and CD4^+^ cells, followed by CD8^+^ cells. On days 2, 4, 8, and 12, the CD3^+^ cell counts were significantly (*p* < 0.05) lower in the negative control group (3.1, 3.7, 4.6, and 8.1) compared with the birds infected with *Salmonella* Enteritidis alone (14.6, 13.5, 11.0, and 14.7) or those treated with *Limosilactobacillus reuteri* (12.2, 13.8, 14.8, and 14.4), *Ligilactobacillus salivarius* (21.3, 18.1, 22.4, and 15.6), *Lactobacillus acidophilus* (12.6, 24.3, 42.8, and 49.3), cecal microbiota (17.5, 22.5, 29.8, and 46.2), or combinations of these treatments with *Salmonella* Enteritidis infection [60].

On day 12, significant (*p* < 0.05) differences in the mean CD4^+^ cell counts were observed in the lamina propria, with values of 3.9 in the negative control group; 6.6 in the positive control group; and 6.8, 7.0, 6.9, and 6.8 in the groups treated with *Limosilactobacillus reuteri*, *Ligilactobacillus salivarius*, *Lactobacillus acidophilus*, and cecal microbiota, respectively, or their combinations with *Salmonella* Enteritidis infection. The CD8^+^ cell counts were highest in the duodenums of birds treated with cecal microbiota and challenged with *Salmonella* Enteritidis, followed by the jejunum and cecum, indicating strong T-cell stimulation [60].

Similarly, in a study that evaluated the immunomodulatory effects of a *Lactobacillus*-based probiotic (LBP) on *Salmonella* Enteritidis infection in layer chickens, birds infected with *Salmonella* Enteritidis alone had significantly (*p* < 0.05) higher CD8^+^ T-cell counts in the cecal tonsils (31.3%) compared with those treated with LBP alone (11.3%) or in combination with *Salmonella* Enteritidis (17.4%) [49]. Conversely, the CD4^+^ T-cell counts in the cecal tonsils were significantly (*p* < 0.05) higher in the LBP (31.3%) and LBP–*Salmonella* Enteritidis (29.5%) groups compared with the *Salmonella* Enteritidis-only group (19.7%), underscoring lactobacilli’s role in mitigating cytotoxic immune responses and reducing gut inflammation [49].

Overall, various lactobacilli strains can stimulate the gastrointestinal immune system by enhancing CD3^+^, CD4^+^, and CD8^+^ lymphocyte infiltration into the intestinal epithelium and lamina propria [60]. The intestinal T-cell population composition is influenced by exposure to luminal antigens, which initiate immune responses through intraluminal stimulation [81].

### 3.3. Fermented Feed’s Effect on Intestinal Morphology

The architecture of the intestinal mucosa provides essential insights into overall gut health [41]. Digestion and nutrient absorption primarily occur at the level of the intestinal villi and crypts, where longer villi and shallower crypts result in an increased surface area for absorption, directly enhancing the growth performance. Key morphological parameters, including the VH, CD, and VH/CD ratio, are recognized as reliable indicators of gut health and performance [82].

In addition to facilitating nutrient uptake, intestinal villi act as natural barriers, preventing the entry of toxic substances and pathogenic bacteria into the intestinal lumen [83]. However, exposure to dietary stressors, such as ANFs, pathogens, and toxins, can adversely affect the intestinal mucosal morphology, leading to shortened villi and deepened crypts. These changes are indicative of increased mucosal turnover and can significantly impair gut health and overall performance [41]. Crypts are key sites for cell proliferation, and deep crypts are often associated with rapid tissue turnover.

Fermented feed has been shown to promote both growth performance and intestinal histomorphology in broilers, likely due to an enhanced nutrient content and bioavailability, including increased polysaccharide, peptide, amino acid, and vitamin levels. These improvements may enable IECs to access nutrients more efficiently and at higher concentrations, supporting epithelial renewal and function [47].

*Aspergillus niger* is a widely used poultry probiotic known for its ability to produce multiple enzymes, including hemicellulases, hydrolases, pectinases, proteases, amylases, lipases, and tannases [84]. Similarly, the leaves of *Ginkgo biloba*, a traditional Chinese herb, possess antimicrobial and anti-inflammatory properties and are used prophylactically against heart disease and certain cancers due to their high flavonoid and terpenoid contents [85,86,87]. To evaluate the effects of *Aspergillus niger*-fermented *Ginkgo biloba* leaves (FR) on broiler growth and intestinal morphology, birds were randomly assigned to five groups: a control group that received a basal diet and four experimental groups [41]. The experimental groups included birds fed a basal diet supplemented with unfermented *Ginkgo biloba* leaves (NF) at 0.35 and 0.7% for starter and grower feeds, respectively, and birds fed a basal diet supplemented with fermented *Ginkgo biloba* leaves at inclusion levels of 0.2, 0.35, and 0.5% in the starter feeds and 0.4, 0.7, and 1.0% in the grower feeds for treatment groups FR1, FR2, and FR3, respectively. By day 42, duodenal VH was significantly (*p* < 0.05) increased in birds supplemented with fermented *Ginkgo biloba* leaves, with VH values of 1689, 1679, and 1684 µm for FR1, FR2, and FR3, respectively, compared with 1558 µm in the control group [41]. The jejunal VH was also significantly (*p* < 0.05) greater in the FR2 group (1295 µm) compared with the control (1141 µm) and NF group (1162 µm). The jejunal CD decreased significantly (*p* < 0.05) in FR1, FR2, and FR3 by 14.3, 16.1, and 14.4 µm, respectively, compared with the control, and by 10.8, 12.6, and 10.6 µm, respectively, compared with the NF group. Additionally, the jejunal VH/CD in the FR2 and FR3 groups increased significantly (*p* < 0.05) by 23.7 and 19.3%, respectively, compared with the control, and by 19.4 and 15.2%, respectively, compared with the NF group [41].

*Bacillus* strains are commonly used as probiotics in poultry feeds due to their ability to form spores, which withstand harsh environmental conditions during processing and in the GIT [88,89]. These endospores germinate in the gut, conferring their probiotic effects on the host [88]. To examine the fermented feed effects produced through the solid-state fermentation of SBM with surfactin-producing *Bacillus* strains (i.e., *Bacillus subtilis* LYS1, *Bacillus subtilis* Lo6, *Bacillus subtilis* NSN7, *Bacillus subtilis* var. *natto* N21, *Bacillus subtilis* N12, and *Bacillus amyloliquefaciens* Da16) on broiler growth and gut morphology, birds supplemented with the fermented product exhibited significantly (*p* < 0.05) longer duodenal and jejunal VHs (1476 and 1445 µm, respectively) compared with the unfermented group (1285 and 1305 µm, respectively) [90]. The CDs in the duodenum and jejunum decreased significantly (*p* < 0.05) in the fermented group (332 and 243 µm, respectively) compared with the unfermented group (526 and 305 µm, respectively). These changes resulted in significant (*p* < 0.05) increases in the VH/CD ratio in the duodenum and jejunum, with values of 93.2 and 40.6%, respectively, in the fermented group compared with the unfermented group [90].

Some studies have not shown significant changes in gut morphology when using fermented feed or feed by-products. However, broilers fed moist fermented feed during the finishing period exhibited significant (*p* < 0.05) increases in the VH by 23 and 16% in the mid-jejunum and mid-ileum, respectively, compared with the control group fed dry unfermented feed [10].

Broiler diet supplementation with plant-derived essential oils, alone or in combination with *Bacillus*-fermented by-products (synbiotics), has been reported to improve growth performance, reduce gut infections and inflammation, and regulate gut microbiota [91,92]. However, a study that evaluated the effects of these synbiotics, either alone or combined with essential oils, on growth performance and gut morphology found no significant differences in the VH or VH/CD ratios across all intestinal segments compared with the control group that received no supplementation [93]. Interestingly, the CD in the duodenum was significantly (*p* < 0.05) higher in birds supplemented with synbiotics alone (208.3 µm) compared with the control (173.4 µm) [93].

Similarly, in a study that investigated the effects of fermented or unfermented palm kernel cake on nutrient digestibility, gut morphology, and gut microflora in broilers, no significant (*p* > 0.05) differences in the VH were observed across all intestinal segments between the birds fed fermented or unfermented palm kernel cake [9].

Fermentation is known to enhance the concentration, digestibility, and bioavailability of nutrients, promoting their more efficient uptake by IECs. Additionally, fermentation produces beneficial microbial metabolites that support the maintenance of intestinal architecture and function [94]. ANFs, such as tannins, phytates, and gossypol, can compromise gut integrity by inducing inflammation and mucosal damage [77]. Fermentation processes significantly reduce the concentrations of these ANFs, thereby exerting protective effects and promoting improved intestinal morphology [95].

Fermented feeds are also rich in LAB, which contribute to gut health. Studies have demonstrated a significant positive correlation (*r* = 0.5) between increased duodenal VH/CD ratios and elevated LAB concentrations in the gut [82].

### 3.4. Fermented Feed’s Effect on Gut Microbiota Composition and Function

Broiler performance has markedly improved due to advancements in nutritional management and a deeper understanding of avian physiology under intensive commercial production systems [96]. Beyond conventional approaches focusing on diet formulation and nutrition optimization, recent research has highlighted the significance of GIT functionality, particularly the role of GALT and gut microbiota, in enhancing broiler performance. Technological advancements, such as high-throughput gene sequencing, have substantially expanded our understanding of GIT microbial ecology, providing insights into how these microbial communities influence broiler health and performance [96]. The intestinal microbiota plays a central role in regulating host nutrition, physiology, gut morphology, and immune function. Fermented feed use supports poultry health by lowering the intestinal pH, promoting lactobacilli proliferation, and modulating the gut microbiota composition, thereby influencing the host’s immune response [26].

Microbial colonization within the avian GIT varies by segment. The crop, proventriculus, and gizzard—regions characterized by a low pH—are predominantly inhabited by *Lactobacillus* species. In contrast, the small intestine contains a lower microbial density and diversity, typically including *Escherichia coli*, *Lactobacillus*, *Enterococcus*, *Helicobacter*, and certain *Clostridium* species [97].

The cecum exhibits the highest microbial concentration and diversity, with its microbiota dominated by two major and two minor phyla. The major phyla, constituting 45% of the total cecal microbes, include Gram-positive Firmicutes (e.g., *Ruminococcaceae* and *Lachnospiraceae*) and Gram-negative Bacteroidetes (e.g., *Rikenellaceae* and *Bacteroidaceae*). The minor phyla, accounting for 2–3% of the cecal microbiota, include Gram-positive Actinobacteria and Gram-negative Proteobacteria, such as *Sutterella* and *Parasutterrella* [97]. Among these groups, the *Bacteroidaceae*, *Lachnospiraceae*, and *Ruminococcaceae* family members are the most commonly identified microbes in chicken cecum, highlighting their critical role in gut health and functionality [97].

Understanding the interactions between pathogenic and non-pathogenic gut microbes has inspired the use of feed additives, such as prebiotics, probiotics, organic acids, and botanicals, into poultry diets [98]. These additives aim to not only limit disease but also enhance productivity [98,99].

Through mechanisms such as competitive exclusion and antimicrobial substance production, various species of LAB can limit or prevent colonization and intestinal infections caused by enteric pathogens [98]. Broilers fed *Lactobacillus*-fermented feed exhibited significantly (*p* < 0.05) higher cecal lactobacilli concentrations, as well as reduced intestinal colonization and fecal shedding of *Salmonella* spp. and coliforms [11]. Factors such as the organ type and location; age; antimicrobials used; and, more specifically, nutrition are known to influence the gut microbiota composition and concentration [98].

The composition and abundance of GIT microbiota are influenced by dietary inputs, with any alteration in feed likely to impact the microbial community structure [20]. Fermented feed affects the gut microbiota through several mechanisms, including a high LAB concentration, elevated lactic acid and SCFA levels, a reduced pH, improved nutrient digestibility, decreased ANF levels, and the presence of prebiotic compounds [38]. LAB, particularly *Lactobacillus* species, exhibit broad-spectrum antagonistic activities against various pathogenic and spoilage microorganisms [100]. This antagonism is largely attributed to their metabolic capacity to convert lactose and other carbohydrates into lactic acid, thereby lowering the gut pH and creating inhospitable conditions for harmful microbes.

In addition to their antimicrobial effects, lactobacilli possess immunomodulatory properties through their interactions with IECs. These interactions facilitate the exchange of immunological signals between the GIT and systemic immune organs, contributing to host immune regulation [101]. This dual role in microbial inhibition and immune modulation makes LAB ideal starter cultures for food and feed product fermentation intended for human and animal consumption.

Research on fermented feed application in poultry remains relatively limited [10], resulting in inconsistent conclusions regarding its impact on broiler gut microbiota. To investigate the effects of feeding solid-state fermented RSM on the intestinal ecology in broilers, birds were divided into three groups: a control group and two experimental groups. In the experimental groups, the control diet was supplemented with 10% of a basal substrate composed of 75% RSM, 24% wheat bran, and 1% brown sugar, either unfermented or fermented using *Limosilactobacillus fermentum*, *Enterococcus faecium*, and *Saccharomyces cerevisiae*. On days 21 and 42, the broilers fed fermented RSM demonstrated significantly (*p* < 0.05) higher cecal and colonic lactobacilli counts compared with those fed the control diet or unfermented RSM [16].

Similarly, to determine the effects of *Bacillus subtilis*-fermented cottonseed meal on broiler growth and cecal microbial populations, SBM replacement with fermented cottonseed meal at 0, 4, 8, and 12% inclusion levels significantly (*p* < 0.01) increased the cecal lactobacilli counts on day 21 to 8.24, 8.75, 9.03, and 8.65 log_10_ colony-forming units (CFU)/g of digesta and on day 42 to 8.51, 8.92, 9.01, and 9.00 log_10_ CFU/g of digesta, respectively [18]. The replacement also significantly (*p* < 0.01) reduced the cecal coliform counts on day 21 to 6.71, 6.49, 6.08, and 6.04 log_10_ CFU/g of wet digesta and on day 42 to 7.40, 7.36, 7.30, and 7.34 log_10_ CFU/g of digesta, respectively [18]. These results support prior evidence indicating that *Bacillus subtilis* enhances gut health by suppressing pathogenic bacteria and promoting beneficial microbial populations [102].

Broilers fed liquid fermented feed showed a significant (*p* < 0.05) reduction in coliform and *Streptococcus* counts in the ileum on day 26 (3.5 and 7.0 log_10_ CFU/mL) compared with controls (4.2 and 7.7 log_10_ CFU/mL). On day 39, coliform counts were also lower in the ileum, crop, and proventriculus/gizzard (5.1, 4.6, and 3.3 vs. 7.0, 6.5, and 5.1 log_10_ CFU/mL). Liquid fermented feed supplementation further reduced the cecal *Streptococcus* counts (7.4 vs. 8.4 log_10_ CFU/mL) and increased the *Lactobacillus* levels in the ileum (8.1 vs. 6.9 log_10_ CFU/mL) and proventriculus (7.5 vs. 6.5 log_10_ CFU/mL) [10].

In another study, liquid fermented feed produced using *Ligilactobacillus salivarius* significantly reduced the fecal shedding duration of *Salmonella enterica* subsp. *enterica* serovar Typhimurium by 53% compared with only an 8% reduction in the control group. Additionally, birds that received liquid fermented feed showed significantly (*p* < 0.05) higher mean lactobacilli counts on day 39 (8.3 log_10_ CFU/mL) compared with the control (7.0 log_10_ CFU/mL) [103].

On day 21, broilers fed 15% *Paenibacillus polymyxa*-fermented palm kernel cake demonstrated significantly (*p* < 0.05) lower gut *Enterobacteriaceae* counts (4.03 log_10_ CFU/mL) compared with birds that received 0–15% unfermented palm kernel cake (4.19–4.27 log_10_ CFU/mL). Moreover, LAB counts in the fermented palm kernel cake group were significantly (*p* < 0.05) higher on day 42 (5.56 log_10_ CFU/mL) compared with the control (5.17 log_10_ CFU/mL) [9].

In laying hens, fermented feed supplementation significantly (*p* < 0.05) reduced the coliform counts in the crop, gizzard, and ileum to 3.64, 3.25, and 5.15 log_10_ CFU/g of digesta, respectively, compared with 5.15, 3.71, and 5.95 log_10_ CFU/g in the control group [104].

Total bacterial DNA extracted from cecal microbiota was used to generate microbial operational taxonomic units (OTUs) through numerical taxonomy. In a study that involved six broiler groups—including a control group fed a basal diet and five treatment groups received either 10–25% dry fermented feed or 10% wet fermented feed fermented with *Lactiplantibacillus plantarum*, *Bacillus subtilis*, and *Saccharomyces cerevisiae*—bacterial DNA sequencing revealed 1389 OTUs shared across all the groups [26]. However, 356, 438, 612, 432, 437, and 595 OTUs were unique to the control; 10, 15, 20, and 25% dry fermented feed; and 10% wet fermented feed groups, respectively. The relative abundances of *Ruminococcaceae*, *Lactobacillaceae*, and unclassified *Clostridiales* were significantly (*p* < 0.05) higher in the cecal microbiota of birds fed either dry or wet fermented feed compared with the control. Conversely, the abundances of *Rikenellaceae*, *Lachnospiraceae*, and *Bacteroidaceae* were reduced in birds that received 15% dry fermented feed. These findings support the conclusion that fermented feed can effectively modulate the microbial composition of the broiler cecum [26].

In summary, evidence from the reviewed literature indicates that fermented feed benefits broiler gut health by improving the intestinal morphology, regulating the immune function, and modifying the gut microbiota. These changes contribute to enhanced nutrient absorption, increased disease resistance, and improved growth performance.

## 4. Conclusions and Future Prospects

Feed fermentation offers a promising strategy to enhance growth performance, gut health, and microbiota composition in broiler chickens. This review highlights the multifaceted fermented feed benefits, including improved nutrient bioavailability, a reduction in ANFs, and the modulation of gut morphology and immunity. The integration of specific *Lactobacillus*, *Bacillus*, and *Saccharomyces* strains into fermentation processes has demonstrated significant improvements in the VH, VH/CD ratio, and microbial diversity in the gut, all of which contribute to better nutrient absorption and reduced pathogen colonization.

However, the variability observed in the FI and growth performances across different studies, particularly during the starter and grower phases, points to the need for standardized and optimized fermentation protocols tailored to broiler production systems. A deeper understanding of microbial dynamics and feed substrate variability is crucial, because uncontrolled fermentation may lead to pathogenic organism proliferation and harmful metabolite accumulation. To ensure product safety and consistency, the use of authenticated microbial strains from the World Federation for Culture Collections-affiliated culture banks is recommended.

Future research should aim to refine fermentation methodologies, elucidate strain-specific probiotic mechanisms, and explore synergistic interactions with prebiotics, enzymes, and phytogenic compounds. Employing multi-omics approaches may provide deeper insights into host–microbe interactions. In the long term, fermented feed holds great potential to sustainably enhance broiler health and performance, reduce the reliance on antibiotics, and support more resilient and efficient poultry productions systems.

## Figures and Tables

**Table 1:** Fermented feed’s effects on broiler chicken growth performance.

Type of Feed	Ingredients	Fermentation Technique	Inoculant	Breed	Age of Birds and Study Duration (Days)	Effect	Reference
Corn–SBM compound feed (moist mash)	Whole diet (100%)	LSF	*Lactiplantibacillus plantarum*	Ross 308	Starter (13) Grower (26) Study (39)	Reduced FI for starters (44%) and growers (23%). Reduced daily WG (11%) and final BW (11%). Improved FCR in finisher phase (9%) and total period (5%).	[10]
Corn–SBM compound feed (moist mash)	Whole diet (100%)	LSF	None (spontaneous fermentation)	Marshall	Grower (14) Study (42)	Increased daily WG (19%) and final BW (18%). No effect on daily FI and FCR.	[14]
Corn–SBM compound feed (dry pellet)	Whole diet (100%)	LSF	*Bacillus subtilis* var. *natto*, *Bacillus coagulans*, *Lactobacillus acidophilus*, *Lactobacillus delbrueckii*, *Lacticaseibacillus casei*, and *Limosilactobacillus reuteri*	Arbor Acres	Finisher (35) Study (35)	Reduced FCR (7%) for feed fermented with *Bacillus* spp. only. Increased WG (7%) for *Bacillus*-fermented pelleted feed.	[15]
Corn–SBM with inclusion of fermented RSM (dry pellet)	Fermented RSM inclusion at 10%	SSF	*Limosilactobacillus fermentum*, *Enterococcus faecium*, *Bacillus subtilis*, and *Saccharomyces cerevisae*	Arbor Acres	Grower (42) Study (42)	Increased WG (7%) and reduced FCR (3%) in broilers fed fermented RSM compared with those fed unfermented RSM.	[16]
Corn–SBM with inclusion of fermented or unfermented RSM (dry mash)	Fermented or unfermented RSM inclusion at 0, 5, 10, and 15%	SSF	*Limosilactobacillus fermentum* and *Bacillus subtilis*	Arbor Acres	Grower (21) Finisher (42) Study (42)	Reduced WG (14%) and increased FCR (12%) at 15% inclusion of fermented RSM compared with groups that received 0, 5, or 10% fermented RSM. No effect on WG, daily FI, and FCR at inclusion levels of 0–10%.	[17]
Corn–SBM with inclusion of fermented CSM (dry pellets)	Fermented CSM inclusion at 0, 4, 8, and 12%	SSF	*Bacillus subtilis*	Yellow-feathered broilers	Starter (0–21) Finisher (22–42) Study (42)	Increased WGs of 3 and 4% at fermented CSM inclusion levels of 4 and 8%, respectively, compared with control (0% inclusion).	[18]
Corn–SBM with inclusion of fermented or unfermented CSM (dry pellets)	Fermented or unfermented CSM inclusion at 0, 10, and 20%	SSF	*Bacillus subtilis*, *Aspergillus oryzae*, and *Aspergillus niger*	Ross 308	Finisher (42) Study (42)	Improved FI, WG, and FCR compared with broilers fed unfermented CSM.	[19]
Corn–SBM with inclusion of fermented or unfermented RSM (dry pellets)	SBM replaced with fermented or unfermented RSM at 50 or 100%	SSF	*Lactobacillus acidophilus*, *Bacillus subtilis*, and *Aspergillus niger*	Ross 308	Finisher (42) Study (42)	50% fermented RSM increased WGs by 6 and 24% and reduced FCRs by 6 and 18% compared with 50 and 100% unfermented RSM, respectively.	[20]
Corn–SBM with SBM replaced with fermented or unfermented RSM (dry pellets)	SBM replaced with unfermented RSM at 0 or 5% or fermented RSM at 5 or 10%	SSF	*Lactobacillus* sp., *Bacillus licheniformis*, and *Candida utilis*	Yellow-feathered broilers	Finisher (42) Study (42)	10% fermented RSM increased average daily WGs by 5 and 10% compared with 0 and 5% unfermented RSM, respectively. 10% fermented RSM reduced FCR by 7% compared with 5% unfermented RSM.	[21]
Corn–SBM with SBM replaced with fermented or unfermented DORB (dry pellets)	Inclusion of fermented or unfermented DORB at 8% in basal diet	SSF	*Saccharomyces cerevisiae*	Cobb 500	Finisher (42) Study (28)	8% fermented DORB increased the WG by 5% compared with control.	[22]

SBM: soybean meal; RSM: rapeseed meal; CSM: cottonseed meal; DORB: de-oiled rice bran; LSF: liquid-state fermentation; SSF: solid-state fermentation; FI: feed intake; WG: weight gain; BW: body weight; FCR: feed conversion ratio.

## Data Availability

The data presented in this article are freely accessible and were obtained from the cited published articles.

## Abbreviations

ANF	anti-nutritional factor
BoF	bursa of Fabricius
BW	body weight
DDGS	distillers’ dried grains with solubles
FCR	feed conversion ratio
FFA	fermented feed additive
FI	feed intake
GALT	gut-associated lymphoid tissue
GIT	gastrointestinal tract
IEC	intestinal epithelial cell
IFN	interferon
IL	interleukin
LAB	lactic acid bacteria
LBP	*Lactobacillus*-based probiotic
LFP	*Bacillus licheniformis*-fermented product
OTU	operational taxonomic unit
RSM	rapeseed meal
SBM	soybean meal
SCFA	short-chain fatty acid
SFP	*Bacillus subtilis*-fermented product
TGF-β	transforming growth factor-beta
TLR	toll-like receptor
TNF	tumor necrosis factor
VH	villus height
VH/CD	villus height-to-crypt depth
WG	weight gain
